# Accuracy of Parent-Reported Child Height and Weight and Calculated Body Mass Index Compared With Objectively Measured Anthropometrics: Secondary Analysis of a Randomized Controlled Trial

**DOI:** 10.2196/12532

**Published:** 2019-09-16

**Authors:** Li Kheng Chai, Clare E Collins, Chris May, Carl Holder, Tracy L Burrows

**Affiliations:** 1 School of Health Sciences Faculty of Health and Medicine The University of Newcastle Callaghan Australia; 2 Priority Research Centre in Physical Activity and Nutrition The University of Newcastle Callaghan Australia; 3 Hunter Medical Research Institute New Lambton Australia; 4 Family Action Centre The University of Newcastle Callaghan Australia

**Keywords:** telemedicine, parents, child, body height, body weight, body mass index, self report, dimensional measurement accuracy

## Abstract

**Background:**

Electronic health (eHealth) interventions for children often rely on parent-reported child anthropometric measures. However, limited studies have assessed parental accuracy in reporting child height and weight via Web-based approaches.

**Objective:**

The objective of this study was to determine the accuracy of parent-reported child height and weight, as well as body mass index and weight category that we calculated from these data. We also aimed to explore whether parent report was influenced by age, sex, weight status, or exposure to participation in a 12-week brief Web-based family lifestyle intervention.

**Methods:**

This study was a secondary analysis of data from a 12-week childhood obesity pilot randomized controlled trial in families with children aged 4 to 11 years in Australia. We asked parents to report demographic information, including child height and weight, using an online survey before their child’s height and weight were objectively measured by a trained research assistant at baseline and week 12. We analyzed data using the Lin concordance correlation coefficient (ρc, ranging from 0 [poor] to ±1 [perfect] concordance), Cohen kappa coefficient, and multivariable linear regression models.

**Results:**

There were 42 families at baseline and 35 families (83%) at week 12. Overall, the accuracy of parent-reported child height was moderate (ρc=.94), accuracy of weight was substantial (ρc=.96), and accuracy of calculated body mass index was poor (ρc=.63). Parents underreported child height and weight, respectively, by 0.9 cm and 0.5 kg at baseline and by 0.2 cm and 1.6 kg after participating in a 12-week brief Web-based family lifestyle intervention. The overall interrater agreement of child body mass index category was moderate at baseline (κ=.59) and week 12 (κ=.54). The weight category calculated from 74% (n=31) and 70% (n=23) of parent-reported child height and weight was accurate at baseline and week 12, respectively. Parental age was significantly (95% CI –0.52 to –0.06; *P*=.01) associated with accuracy of reporting child height. Child age was significantly (95% CI –2.34 to –0.06; *P*=.04) associated with reporting of child weight.

**Conclusions:**

Most Australian parents were reasonably accurate in reporting child height and weight among a group of children aged 4 to 11 years. The weight category of most of the children when calculated from parent-reported data was in agreement with the objectively measured data despite the body mass index calculated from parent-reported data having poor concordance at both time points. Online parent-reported child height and weight may be a valid method of collecting child anthropometric data ahead of participation in a Web-based program. Future studies with larger sample sizes and repeated measures over time in the context of eHealth research are warranted. Future studies should consider modeling the impact of calibration equations applied to parent-reported anthropometric data on study outcomes.

## Introduction

### Background

The wide coverage of the internet and the increase in technology use worldwide have led to the emergence of electronic health (eHealth) for lifestyle interventions [1,2]. Web-based platforms are increasing in popularity and used for data collection and delivery of eHealth interventions [1,3]. In Australia, technology use is increasing and not limited by socioeconomic status or location, with more than 97% of households with children under age 15 years having access to the internet via computer, smartphone, or tablet [4]. Research suggests that online data collection and delivery of interventions is more cost effective than conventional face-to-face modes [5], allows providers to connect with a large number of people simultaneously, and enhances access to services for communities living in rural and remote locations [6].

One limitation, however, is that eHealth interventions and non–face-to-face programs are usually delivered over distance. Hence, interventionists have had to rely on self-reported measures (eg, anthropometrics, diet, physical activity) when objective measurements were not possible. For young children in eHealth lifestyle interventions, these measures often include parent-reported child height and weight data. The risk associated with self-reported height and weight data is that discrepancy with other objective measures can result in miscalculation of weight trajectories and weight category. Parental underestimation of their child’s weight has important clinical implications due to the health consequences of childhood obesity [7] and the importance of early identification of a weight gain trajectory in order to seek early intervention. Misreporting may also influence a child’s actual eligibility for research or treatment programs that recruit participants using self-reported screening surveys.

Studies have used face-to-face interviews or surveys completed at home visits or during clinic visits to collect parent-reported child data. However, data collection may differ between remote non–person-to-person methods (eg, Web-based, posted paper surveys) and direct person-to-person methods (eg, home visits, clinic visits, telephone interviews). Very few studies have evaluated parental accuracy in reporting the height and weight of their children remotely without the presence of clinicians or researchers (ie, online surveys). Furthermore, most of the previous research on parental reporting of their child’s height and weight was conducted in Canada, Western Europe, or the United States [8-12], and no studies have included Australian children. Therefore, it is unknown whether Australian parents would perceive their children’s height and weight in similar ways to parents in other countries. Moreover, previous studies have used limited measures to assess agreement, such as Pearson correlation coefficients or paired *t* tests. These measures were unable to adequately detect levels of agreements (ie, accuracy and precision) and, rather, they are associations between parent-reported and researcher- or clinician-measured anthropometric data only [13].

### Objective

We aimed to determine (1) the accuracy of parent-reported child height and weight, as well as the body mass index (BMI) and weight category that we calculated from these data, compared with data measured objectively by researchers as the reference standard, and (2) whether parent report was influenced by age, sex, weight category, or exposure to participation in a 12-week brief Web-based family lifestyle intervention.

## Methods

### Data Source

This was a secondary analysis of data from a pilot randomized controlled trial that aimed to investigate the feasibility of a 12-week Web-based family lifestyle intervention to support parents in improving their child’s weight status and eating habit [14]. The intervention group received 2 semistructured telehealth consultations (online video consultations, attended by at least one parent and the index child) with Accredited Practising Dietitian, access to the Back2basics Family program website [15], a Facebook group, and additional evidence-based short message service (SMS) text messaging targeted to the parents. The waitlist control group received all the intervention components at 3-month follow-up from baseline.The pilot trial received ethics approval from the Hunter New England Human Research Ethics Committee (16/07/20/4.04), New Lambton, Australia, and University of Newcastle Human Research Ethics Committee (H-2016-0329), Callaghan, Australia. The trial is reported in accordance with the Consolidated Standards of Reporting Trials of Electronic and Mobile Health Applications and Online Telehealth (CONSORT-EHEALTH) checklist (Multimedia Appendix 1 [16]).

### Participants

We recruited families from New South Wales, Australia through clinician referrals, school newsletters, flyers, and word-of-mouth. Eligible families were those who had a child aged 4 to 11 years with a BMI of 21.5 kg/m^2^ or greater (International Obesity Task Force [IOTF] child cutoffs) [17], had access to the internet, and were able to attend laboratory measurement sessions at 1 of the 3 study sites (Newcastle, Tamworth, and Armidale). Parents’ written consents and children’s assents were procured prior to the baseline laboratory measurement session.

### Data Collection

We asked parents to report demographic information of themselves and an identified index child from the family participating in the intervention. Demographic information included age, sex, height, weight, highest education attained (parent only), and postcode (parent only). Parents provided these details using an online survey before their child’s anthropometric measurements were clinically assessed at baseline and week 12, which is a 3-month follow-up from baseline.

We provided no specific instructions to the parents when asked about reporting their child’s height and weight (eg, use a tape or scale or time of day to measure). The questions were “What is your child’s height in cm (if unsure, please estimate)” and “What is your child’s weight in kg (if unsure, please estimate)”. Subsequently, the child’s height and weight were measured at baseline and week 12 using standard protocols by blinded research assistants.

In the laboratory sessions, child height was measured to the nearest 0.1 cm while the child was standing with their head and chin up, looking straight ahead (ie, held in the Frankfurt plane) using the BSM370 Automatic Stadiometer (Biospace Co Ltd, Seoul, South Korea). Child weight was measured to the nearest 0.1 kg without shoes and in light clothing using the 720 body composition analyzer (InBody Co, Ltd, Seoul, South Korea). Measurements were taken twice, and the difference between measures was required to be 0.3 cm or less (height) and 0.4 kg or less (weight). Otherwise, a third reading was obtained, and the 2 closest readings were used to compute an average height or weight measurement. Families were offered an Aus $10 gift voucher for participation in each laboratory measurement session. Analyses were conducted by a researcher not involved in the laboratory measurements.

### Statistical Analysis

All data manipulation and statistical analyses were undertaken using Stata version 12 (StataCorp LLC). We calculated descriptive statistics for baseline participant characteristics. We used parent-reported and researcher-measured child height and weight data to calculate child BMI, with weight category based on IOTF child cutoffs [17]. We used the Lin concordance correlation coefficient (CCC) to assess the level of agreement of parent-reported child height and weight and the calculated BMI compared with researcher-measured data at baseline and week 12. We chose this as a superior method because it measures both precision (ie, Pearson correlation coefficient) and accuracy, thus indicating how well a set of bivariate data compares with the reference standard, measured data. Lin CCC (ρc, ranging from 0 to ±1) is interpreted as almost perfect agreement (ρc>.99), substantial agreement (ρc range .951-.99), moderate agreement (ρc range .90-.950), and poor agreement (ρc<.90) [18]. We used Cohen kappa coefficient to ascertain interrater agreement between the child weight category calculated from parent-reported child height and weight and the researcher-measured data [19]. Cohen kappa coefficient (κ, ranging from 0 to 1) is interpreted as almost perfect (κ>.80), substantial (.61≤κ≤.80), moderate (.41≤κ≤.60), fair (.21≤κ≤.40), slight (.00≤κ≤.20), and poor (κ<.00) [20]. We used multivariable linear regression models for the outcomes of difference between parent-reported and researcher-measured height and weight (calculated by subtracting researcher-measured data from parent-reported data) to further investigate relationships between outcomes and age (years), sex, and weight status of parents and the index child. We calculated a sample size of 7 and 29 participants per group as needed to detect expected correlation coefficients of .9 and .5, respectively, at an alpha of .05 and with 80% power for height and weight.

## Results

### Participant Characteristics

Parent-reported and researcher-measured child height and weight data were available from 42 families at baseline and 35 families (83%) at week 12. Baseline characteristics of children and parents who were lost to follow-up, defined as not responding after 3 reminders to complete assessments, were not significantly different from the baseline characteristics of those who completed the follow-up at week 12 [14].

Most parents were female (40/42, 95%) with a mean age 40.5 years and mean BMI 29.9 kg/m^2^, were from middle socioeconomic background (n=28, 67%), and attained a certificate or diploma level of education (n=13, 31%) or a university degree (n=11, 26%). Parents were classified into the obese (n=19, 46%), overweight (n=14, 33%), and healthy weight (n=9, 21%) categories based on the IOTF adult cutoffs [17]. Children were fairly evenly represented by sex (n=24 male, 57%) with a mean age 8.5 years, mean BMI 22.9 kg/m^2^, and weight category of obese (n=22, 52%), overweight (n=9, 21%), and healthy weight (n=11, 26%) based on the IOTF child cutoffs [17]. Table 1 presents detailed participant characteristics.

**Table 1:** Baseline characteristics of parents and their children.

Characteristics		Intervention group (n=28)	Control group (n=14)	Combined (n=42)
**Parents**				
	Age (years), mean (SD)	41 (7)	39 (8)	41 (7)
	Female sex, n (%)	26 (93)	14 (100)	40 (95)
	BMI^a^ (self-reported), mean (SD), kg/m^2^	28.8 (5.2)	32.0 (7.8)	29.9 (6.3)
**Weight category^b^ (self-reported), n (%)**				
	Healthy weight	6 (21)	3 (21)	9 (21)
	Overweight	11 (39)	3 (21)	14 (33)
	Obese	11 (39)	8 (57)	19 (46)
**Education level, n (%)**				
	School certificate	1 (4)	2 (14)	3 (7)
	Higher school certificate	4 (14)	2 (14)	6 (14)
	Certificate or diploma	11 (39)	2 (14)	13 (31)
	Undergraduate degree	7 (25)	4 (29)	11 (26)
	Postgraduate degree	5 (18)	4 (29)	9 (21)
**Socioeconomic status, n (%)**				
	Low (IRSAD^c^ 1-3)	4 (14)	2 (14)	6 (14)
	Mid (IRSAD 4-7)	17 (61)	11 (79)	28 (67)
	High (IRSAD 8-10)	7 (25)	1 (7)	8 (19)
**Children**				
	Age (years), mean (SD)	9 (2)	9 (2)	9 (2)
	Female sex, n (%)	13 (46)	5 (36)	18 (43)
**Anthropometry, mean (SD)**				
	Height (measured), cm	138 (16)	135 (16)	137 (16)
	Weight (measured), kg	44 (17)	46 (19)	45 (17)
	BMI (measured), kg/m^2^	22.4 (4.7)	23.8 (5.9)	22.9 (5.1)

^a^BMI: body mass index.

^b^Weight categories as per International Obesity Task Force age-appropriate cutoffs: healthy weight, BMI 18.5-24.9 kg/m^2^; overweight, BMI 25-29.9 kg/m^2^; obese, BMI ≥30 kg/m^2^.

^c^IRSAD: Index of Relative Socio-Economic Advantage and Disadvantage.

### Agreement Between Parent-Reported and Researcher-Measured Data

**Figure 1:**
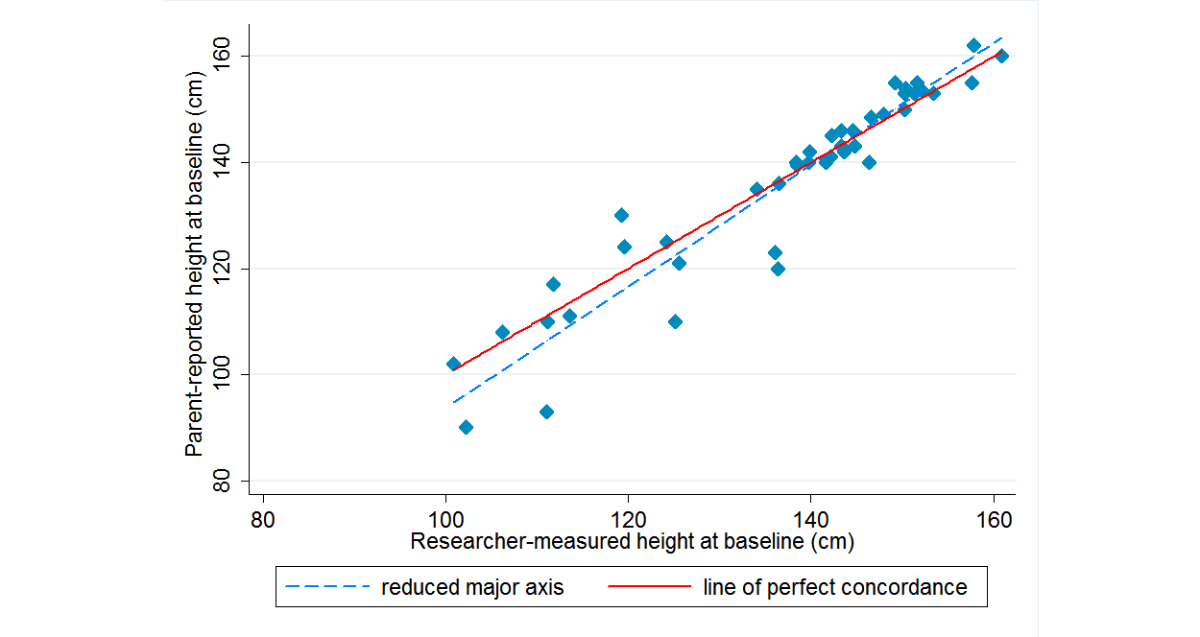
Concordance between parent-reported and researcher-measured height at baseline.

**Figure 2:**
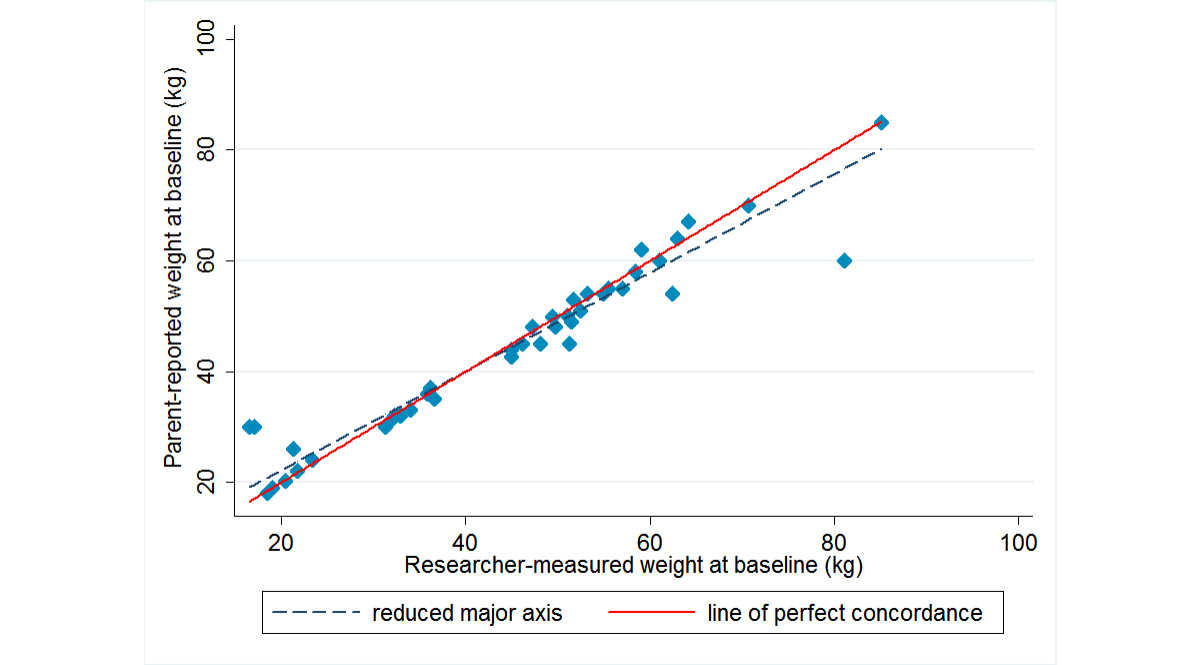
Concordance between parent-reported and researcher-measured weight at baseline.

**Figure 3:**
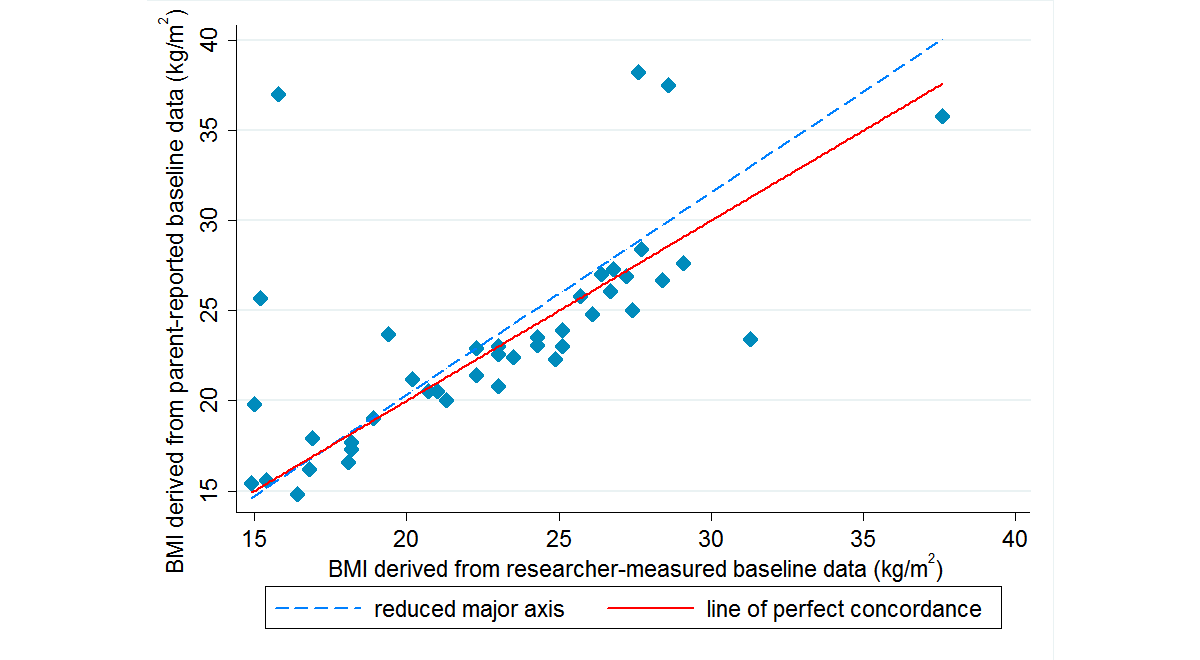
Concordance between body mass index (BMI) calculated from parent-reported data and from researcher-measured data at baseline.

Table 2 summarizes the level of agreement between the parent-reported child height and weight and calculated BMI and the researcher-measured data. At baseline, the level of agreement between parent-reported and researcher-measured data as determined by Lin CCC was moderate (ρc=.94) for parent-reported child height and substantial (ρc=.96) for weight, and poor (ρc=.63) for calculated BMI. In this study, parents tended to underreport their child’s height and weight, with a mean (SD) difference of –0.9 (SD 6.0) cm and –0.5 (SD 4.9) kg, respectively, compared with researcher-measured data. Parents were more accurate in reporting the height of children who were taller than 140 cm (Figure 1) and were overall better reporters of the weight of children who weighed between 30 and 50 kg (Figure 2). BMI calculated using parent-reported data was higher than researcher-measured data with a mean (SD) difference of 0.7 (SD 4.7) kg/m^2^. Figure 3 demonstrates that child BMI calculated from parent-reported data was more accurate (ie, closer to researcher-measured data) for children whose BMI was between 15 and 25 kg/m^2^ compared with those whose BMI was at either end of the spectrum (ie, <15 kg/m^2^ or >25 kg/m^2^).

**Table 2:** Level of agreement between parent-reported and researcher-measured child height, weight, and calculated body mass index (BMI).

Time point		Intervention group			Control group			Combined		
		MD^a^ (SD)	ρc^b^	95% CI	MD (SD)	ρc	95% CI	MD (SD)	ρc	95% CI
**Baseline**		**n=28**			**n=14**			**n=42**		
	Height (cm)	–1.9 (5.7)	.94	0.90-0.98	1.0 (6.3)	.94	0.87-0.99	–0.9 (6.0)	.94	0.91-0.97
	Weight (kg)	–1.1 (5.3)	.94	0.90-0.98	0.7 (4.0)	.98	0.95-1.00	–0.5 (4.9)	.96	0.93-0.98
	BMI (kg/m^2^)	0.7 (4.9)	.53	0.26-0.80	0.7 (4.2)	.76	0.52-1.00	0.7 (4.7)	.63	0.45-0.81
**Week 12**		**n=21**			**n=14**			**n=35**		
	Height (cm)	–0.2 (6.4)	.93	0.87-0.99	–3.9 (8.8)	.86	0.74-0.99	–1.7 (7.5)	.90	0.84-0.96
	Weight^c^ (kg)	–1.6 (3.3)	.98	0.95-1.00	–2.9 (2.6)	.98	0.96-1.00	–2.1 (3.0)	.98	0.96-0.99
	BMI^c^ (kg/m^2^)	–0.9 (2.3)	.85	0.73-0.98	0.2 (3.6)	.84	0.68-1.01	–0.5 (2.9)	.86	0.76-0.95

^a^MD: mean difference (parent-reported value subtract researcher-measured value).

^b^ρc: Lin concordance correlation coefficient, ranging from 0 to ±1, where a value close to 1.0 (and a 45° fitted line of perfect concordance) suggests a perfect level of agreement, .951-.99 is substantial agreement, .90-.950 is moderate agreement, and <.90 is poor agreement).

^c^Data were available from n=19 intervention families due to missing parent-reported weight.

At week 12, the level of agreement between parent-reported and researcher-measured child height remained moderate (ρc=.93) in the intervention group but declined from moderate to poor agreement (ρc=.86) in the control group. Parents’ accuracy in reporting their child’s weight improved in the intervention group, demonstrating substantial agreement (ρc=.98) with researcher-measured data, whereas control group parents remained at the same level of agreement as at baseline (ρc=.98). At week 12, parents in the intervention group continued to underreport their child’s height and weight with a mean difference (SD) of –0.2 cm (SD 6.4) and –1.6 kg (SD 3.3), respectively, compared with researcher-measured data. However, these parents demonstrated improved accuracy in reporting their child’s height (–0.9 cm to –0.2 cm), but not in reporting their child’s weight (–0.5 kg to –1.6 kg). Parents in the control group overreported their child’s height and weight at baseline by 1 cm (SD 6.3) and 0.7 kg (SD 4.0), respectively. However, at week 12 the control group parents also underreported their child’s height and weight and to a greater extent than the intervention group, and –3.9 cm (SD 8.8) and –2.9 kg (2.6), respectively, lower than researcher-measured data. At week 12, the level of agreement for calculated BMI using parent-reported and researcher-measured data improved but remained poor for both the intervention group (ρc=.85 vs ρc=.53) and the control group (ρc=.84 vs ρc=.76). However, the mean differences of parent-reported and researcher-measured height, weight, and BMI calculated from these data at baseline and week 12 were not statistically significant between the intervention and control groups.

### Interrater Agreement

Table 3 summarizes interrater agreement of child weight category based on calculated BMI using parent-reported and researcher-measured child height and weight. At baseline, the level of agreement was moderate (κ=.59). Overall, the weight category (ie, healthy weight, overweight, obese) calculated from 74% (31/42) of parent-reported child height and weight at baseline was accurate (ie, within the same category calculated based on objectively measured data). Of the 11 children in the healthy weight category, the weight category calculated from 55% (6/11) of parent-reported data was accurate, whereas the weight category calculated from 45% (5/11) of parent-reported data misclassified their child in the overweight (3/11, 27%) or obese (2/11, 18%) category. Of the 9 children in the overweight category, the weight category calculated from 89% (8/9) of parent-reported data was accurate, whereas 11% (1/9) of parent-reported data misclassified children who were overweight into the healthy weight category. Among the 22 children in the obese category, the weight category calculated from 77% (17/22) of parent-reported data was accurate, whereas 23% (5/22) of parent-reported data misclassified children as overweight. At week 12, the interrater agreement for child weight category decreased in both the intervention (71% vs 68%) and control groups (79% vs 71%). The level of agreement remained moderate in the intervention (κ=.54) and control groups (κ=.51), as well as for both groups combined (κ=.54). Among the children in the healthy weight category, the weight category calculated from 100% (5/5) of parent-reported data in the intervention group (vs 2/3, 67% in the control group) was accurate at week 12. Among children who were overweight, the weight category calculated from 67% (4/6 and 2/3) of parent-reported data in the intervention and control groups was accurate at week 12, respectively. Among children who were obese, the weight category calculated from 50% (4/8) of parent-reported data in the intervention group (vs 75% (6/8) in the control group) was accurate.

In all families except 1, the same parent reported child height and weight at both time points. Overall, the weight category calculated from 55% (23/42) of parent-reported data was accurate (ie, closer to researcher-measured data) at both the baseline and week 12 time points. The weight category calculated from a small number of parent-reported data was consistently 1 category under (3/42, 7%) or above (1/42, 2%) their child’s correct weight category at both time points. Further analysis did not find statistically significant differences in demographic characteristics (eg, age and sex of parent and child, parental BMI, education, and socioeconomic status) between underreporters and overreporters. A multivariable linear regression model identified that parental age was the only variable that had a significant association (*P*=.01) with accuracy of reporting child height. Every 1-unit (in years) increase in the parent’s age resulted in an underreporting of 0.29 cm (95% CI –0.52 to –0.06; *P*=.01) for child height. Child age was the only variable that was significantly associated with reporting of child weight. Every 1-unit (in years) increase in the child’s age resulted in an underreporting of 1.2 kg (95% CI –2.34 to –0.06; *P*=.04) for child weight.

**Table 3:** Interrater agreement for child weight category^a^ calculated using parent-reported versus researcher-measured child height and weight.

Time point and parent-reported data		Calculated from researcher-measured data							
		Intervention group^b^				Control group^c^			
		Healthy weight	Overweight	Obese	Total	Healthy weight	Overweight	Obese	Total
**Baseline, n (%)**		**n=28**				**n=14**			
	Healthy weight	4 (14)	1 (4)	0 (0)	5 (18)	2 (14)	0 (0)	0 (0)	2 (14)
	Overweight	3 (11)	6 (21)	3 (11)	12 (43)	0 (0)	2 (14)	2 (14)	4 (29)
	Obese	1 (4)	0 (0)	10 (36)	11 (39)	1 (7)	0 (0)	7 (50)	8 (57)
	Total	8 (29)	7 (25)	13 (46)	28 (100)	3 (21)	2 (14)	9 (64)	14 (100)
**Week 12, n (%)**		**n=19^d^**				**n=14**			
	Healthy weight	5 (26)	2 (21)	0 (0)	7 (37)	2 (14)	0 (0)	0 (0)	2 (14)
	Overweight	0 (0)	4 (21)	4 (21)	8 (42)	0 (0)	2 (14)	2 (14)	4 (29)
	Obese	0 (0)	0 (0)	4 (21)	4 (21)	1 (7)	1 (7)	6 (43)	8 (57)
	Total	5 (26)	6 (32)	8 (42)	19 (100)	3 (21)	3 (21)	8 (57)	14 (100)

^a^Weight categories as per International Obesity Task Force age-appropriate cutoffs: healthy weight, body mass index (BMI) 18.5-24.9 kg/m^2^; overweight, BMI 25-29.9 kg/m^2^; obese, BMI ≥30 kg/m^2^.

^b^Baseline: Cohen kappa coefficient= .57, SE 0.13, and % agreement=71; week 12: Cohen kappa coefficient=.54, SE 0.15, and % agreement=68 (Cohen kappa coefficient ranges from 0 to 1, where κ>.80 is almost perfect, .61≤κ≤.80 is substantial, .41≤κ≤.60 is moderate, .21≤κ≤.40 is fair, .00≤κ≤.20 is slight, and κ<.00 is poor).

^c^Baseline: Cohen kappa coefficient=.62, SE 0.19, and % agreement=79; week 12: Cohen kappa coefficient=.51, SE 0.19, and % agreement=71.

^d^Data were available from 19 intervention families due to missing parent-reported weight.

## Discussion

### Principal Findings

We evaluated the accuracy of online parent-reported child height and weight, as well as BMI and weight category that we calculated from these data, compared with researcher-measured data in a sample of Australian children aged 4 to 11 years. The study also examined whether accuracy of parental-reporting was influenced by age, sex, BMI, and participation in a 12-week brief Web-based family lifestyle intervention.

Key findings indicated that parents were relatively accurate in reporting their child’s height and weight as shown by the overall high CCCs (ρc≥.9). Results indicated that parents in this study underreported their child’s height and weight at both baseline and after participating in a 12-week Web-based family lifestyle intervention. These findings were similar to previous studies that indicated that parents tended to underreport the height and weight of American children aged 6 to 12 years [21]. Previous studies found that parents underreported their child’s height and weight by –1.4 cm and –2.3 kg, respectively (n=475 American children aged 11-12 years) [22], and by –1 cm and –1.6 kg, respectively (n=116 Belgian children aged 7-9 years) [23]. This compares with a study in 662 children in the United States, which found that 35% of parents underreported their child’s height by at least 1 inch (2.54 cm) and 26% by at least 2 inches (5.08 cm) [24]. In that study, 22% of parents of children aged 3 to 5 years (n=343) and 39% of parents of children aged 6 to 12 years (n=452) underestimated their child’s weight by at least 2 lbs (0.9 kg) [24]. It is evident across previous research that parents’ inaccuracy in reporting their child’s height and weight, though varying in extent and by country, was commonly due to underreporting instead of overreporting, regardless of the measurement systems used (metric vs imperial system).

Previous studies have highlighted that parents were more likely to underreport their child’s height than their weight [21,24]. Our study arrived at similar findings, where parents underreported their child’s height and weight, and were generally less accurate in reporting their child’s height (ρc range .86-.94) than their weight (ρc range .94-.98) as demonstrated by the consistently higher CCC for child weight over time. This suggests that children may be weighed more regularly or accurately than they are measured for height. It is possible that parents measured their child’s weight at home using a weighing scale, which is a common household item. Furthermore, enrolling in a weight management program may make them more aware of their child’s weight than their height. In contrast, child height may not be measured as regularly due to not having a stadiometer at home, or as accurately due to not using the Frankfurt plane position, which is the standard protocol for measuring height. However, there are discrepancies between this study and a previous study that found poor concordance between parent-reported and researcher-measured child height (ρc=.007), weight (ρc=–.039), and BMI (ρc=–.005) [13]. It was suggested that a sample of parents in California, USA may have been inclined to report child height in whole inches, resulting in a greater degree of underreporting, or overreporting by 2.54 cm [13]. Using the smaller increments of the metric system may therefore enhance parents’ accuracy and precision in reporting their child’s height in centimeters, which is a smaller unit [13]. Due to the differences between the metric and imperial systems, study findings in US populations may not be generalizable to those of countries using the metric measurement system [21].

Despite our finding of a consistent trend in underreporting over time, parents who completed a 12-week brief Web-based family lifestyle intervention demonstrated improved accuracy in reporting their child’s weight (ρc increased from .94 to .98) across time points in the study, whereas the control group maintained their high accuracy from baseline to 12 weeks (ρc remained at .98). Parents may have become more attentive to child weight information received at clinic or intervention visits or from other sources, or may have recorded height and weight measures at home more regularly after participating in the baseline survey and the intervention program. Studies also suggest that parental accuracy in reporting their child’s height and weight may be influenced by whether the parents know that their child’s height and weight will be measured by treating clinicians at a later time [8-10,25], and whether parents were asked to self-measure their child’s height and weight before reporting [26]. Hence, suggesting to parents that their child’s measurements will be validated or providing instructions to parents to measure their child’s height and weight themselves may improve accuracy in parental reporting [13].

The underreported child height and weight in this study resulted in poor concordance (ρc=.86) between BMI calculated from parent-reported and BMI calculated from researcher-measured data. Overall, BMI was underestimated by 0.5 kg/m^2^ when calculated from parent-reported data. Similar findings were reported in other studies in which BMI calculated by researchers from parent-reported child height and weight data was 0.5 kg/m^2^ lower than BMI calculated from objective measures (n=475 children aged 12-13 years) [22]. In another study, BMI was 0.6 kg/m^2^ lower than the BMI calculated from researcher-measured data (n=116 children aged 7 to 9 years) [23]. In this study, we found that child BMI calculated from parent-reported data was more accurate for children whose BMI was between 15 and 25 kg/m^2^ than for those whose BMI was at either end of the spectrum. Similar findings were reported in a study (n=864 Dutch children aged 4 years) in which parents tended to misreport the weight of their children in the lowest and highest BMI quartiles, and the authors suggested that the turning point for overreporting and underreporting of child BMI appeared to be around 15.4 kg/m^2^ [11]. The significance of a misreported BMI depends on whether the BMI is close to the lower or upper range of a weight category. For example, for a 9-year-old boy whose measured BMI is 19 kg/m^2^ (overweight category), an underreport by 0.5 kg/m^2^ would result in a reported BMI of 18.5 kg/m^2^, incorrectly placing the child into the healthy weight category. Studies indicated that misclassification of children as obese based on parent-reported data was associated with underreporting of child height [12,24], as the misreporting was magnified through the BMI calculation formula (ie, weight in kilograms divided by height in meters squared). For this reason, the use of a height or weight percentile might be useful in future studies when interpreting parent-reported child height and weight, instead of calculating BMI to determine child weight category. Future studies may consider modeling a calibration equation for adjusting BMI calculated from parent-reported data to improve accuracy.

Parental underreporting of child height and weight resulted in underestimation of child BMI and misclassification of weight category among 30% (10/33) of children in our study. Overall, child weight category calculated using parent-reported child height and weight at baseline was accurate for 74% (31/42) of families, and this was reduced to 70% (23/33) at week 12. Similar findings were reported in 2 other studies in which child weight category was calculated by researchers using parent-reported child height and weight data, with the BMI category being accurate for 80% (n=558) [24] and 76% (n=600 Austrian children aged 0-15 years) [27]. Among the overweight children in our study, the overall proportion of parents who underestimated their child’s weight category ranged from 11% (1/9) to 22% (2/9) over time. Among children in the obese category, the overall proportion of parents who underestimated their child’s weight category ranged from 23% (5/22) to 38% (6/16) over time. Previous studies regularly reported misclassifications of overweight and obese children [11,27]. One study reported that 46% of 116 overweight children were misclassified as healthy weight when parent-reported data were used [11]. In another study of 600 children aged 0 to 15 years, parents reported that 37% of obese children were incorrectly classified in the overweight category [27]. Such misclassifications, if not addressed and corrected, or accounted for in interpretation, could have an impact on obesity prevalence statistics or intervention programs calculated using parent-reported child height and weight data.

### Strengths and Limitations

A particular limitation of this study was the small sample size, which impeded the modeling of calibration equations to improve the validity of parent-reported data. A large sample would be needed to generate a viable predictive model. Parents tend to be less accurate in reporting for children with excess body weight. Therefore, the study sample comprised children with a BMI above the midpoint of the healthy weight category (≥21.5 kg/m^2^) in order to assess parents’ accuracy in reporting the height and weight of children with a higher weight. This means that our results may not be generalizable or applicable to other populations and ethnicities and, hence, results should be interpreted with caution. The study, although not population based, is to our knowledge the first Australian study to assess parental accuracy in online reporting of their child’s height and weight, and weight status determined by BMI calculated using parent-reported data compared with objective researcher-measured data in a sample of children aged 4 to 11 years. Given that no specific instructions about how to take height and weight measures were provided to parents, a limitation is that parents may or may not have measured their child before reporting the measures. Future studies should explore whether parents’ accuracy in reporting their child’s anthropometrics improves when specific guidance [28] on when and how to perform the measurements is provided. However, it could be challenging to assess parents’ adherence to the specific guidance. Moreover, parents may be less likely to measure a child who is sensitive about weight and body image. Future studies should collect information on whether parent-reported data were based on estimates or measurements, and whether the measurements were done at home, school, or a clinic, to further the understanding of parents’ accuracy in measuring or estimating child height and weight.

A key strength of our study was the use of Lin CCC to assess the level of agreement between parent-reported and researcher-measured data and, hence, offer some confidence in the findings. Many previous studies measured correlations between data, which is insufficient in assessing levels of agreements (ie, accuracy and precision). For example, Pearson correlation coefficients only measure the extent to which the parent-reported data conform to the best-fitting straight line, but not how close or far the data fall from the line that represents perfect agreement [20].

### Conclusions

We found that Australian parents of children aged 4 to 11 years were reasonably accurate in reporting their child’s height and weight online. The weight category for the majority of children calculated using parent-reported data was in agreement with the objectively measured data despite the BMI calculated from parent-reported data having poor concordance at both time points. It appears that online parent-reported child height and weight may be a valid method of collecting child anthropometric data ahead of participation in a Web-based health, diet, and lifestyle program. Future studies with larger sample sizes and repeated measures over time in the context of eHealth research are warranted.

## Appendix

Multimedia Appendix 1 CONSORT-EHEALTH checklist (V 1.6.1).

## Abbreviations

BMI: body mass index
CCC: concordance correlation coefficient
CONSORT-EHEALTH: Consolidated Standards of Reporting Trials of Electronic and Mobile Health Applications and Online Telehealth
eHealth: electronic health
IOTF: International Obesity Task Force
SMS: short message service
